# Verbascoside triggers apoptosis and ferroptosis in NSCLC by targeting BCAT2

**DOI:** 10.1371/journal.pone.0354955

**Published:** 2026-07-30

**Authors:** Ruijuan Li, Bin Li, Ping Wang

**Affiliations:** Department of Respiratory Medicine, The Fourth Hospital of Hebei Medical University, Shijiazhuang, Hebei, China; Marshall University, UNITED STATES OF AMERICA

## Abstract

**Background:**

The treatment of non-small cell lung cancer (NSCLC) has challenges such as drug resistance and recurrence. Concurrently, the induction of apoptosis and ferroptosis is a promising therapeutic strategy. This study aimed to investigate whether the natural product, verbascoside, induces apoptosis and ferroptosis in NSCLC cells by targeting BCAT2.

**Methods:**

Bioinformatic analysis was used to predict the potential targets of verbascosides. Stable cell lines with BCAT2 knockdown and overexpression were constructed. The effects of verbascoside on NSCLC were evaluated *in vitro* and using a mouse xenograft model.

**Results:**

Bioinformatics screening and molecular docking identified BCAT2 as a potential target of verbascoside, with a significantly stronger binding energy (−7.8 kcal/mol) than another candidate, PARP1. *In vitro* and *in vivo* experiments confirmed that BCAT2 knockdown significantly inhibited NSCLC cell viability, induced apoptosis and ferroptosis, and induced mitochondrial damage. Conversely, BCAT2 overexpression produced opposite effects. Verbascoside treatment inhibited BCAT2 expression in a concentration-dependent manner, recapitulating the apoptotic, ferroptotic, and mitochondrial damage phenotypes induced by BCAT2 knockdown; however, BCAT2 overexpression significantly reversed these effects of verbascoside. In an animal model, treatment with verbascoside significantly suppressed tumor growth and activated apoptosis and ferroptosis in tumor tissues by downregulating BCAT2.

**Conclusions:**

Verbascoside can induce apoptosis and ferroptosis in NSCLC by directly targeting and inhibiting BCAT2, leading to mitochondrial dysfunction. This finding not only reveals BCAT2 as a novel target of verbascoside but also confirms its ability to induce apoptosis and ferroptosis in NSCLC cells.

## Background

Lung cancer is the leading cause of cancer-related deaths worldwide, with non-small cell lung cancer (NSCLC) accounting for majority of cases. Despite continuous advances in treatment, drug resistance and recurrence remain severe clinical challenges [1]. Therefore, exploring new strategies that can simultaneously activate multiple cell death pathways is of great significance. Apoptosis, a classic form of programmed cell death, has long been a core target in cancer therapy; however, tumor cells often develop resistance through mechanisms such as upregulation of anti-apoptotic proteins [2]. In recent years, ferroptosis, a novel form of iron-dependent, lipid peroxidation-driven cell death, has garnered significant attention owing to its effectiveness in eliminating tumor cells that are insensitive to apoptosis [3]. Studies indicate that the simultaneous induction of apoptosis and ferroptosis can produce synergistic antitumor effects [4].Owing to their structural diversity and multi-target characteristics, natural products represent a valuable resource for discovering such dual inducers.

Branched-chain amino acid transaminase 2 (BCAT2) has recently emerged as a promising therapeutic target for cancer treatment. Studies have shown that BCAT2 is highly expressed in various cancer tissues, and its expression level is closely associated with poor patient prognosis, suggesting a key role for BCAT2 in lung cancer progression [5,6]. Particularly important is the emerging evidence revealing the direct role of BCAT2 in regulating cell fate: on one hand, BCAT2 may regulate cellular sensitivity to ferroptosis by affecting specific metabolic pathways that interfere with intracellular redox homeostasis; in contrast, alterations in BCAT2 expression or activity may indirectly affect apoptosis-related signaling pathways [5]. These findings position BCAT2 as a potential hub node capable of simultaneously regulating apoptosis and ferroptosis, making it an attractive intervention target for achieving dual death induction through a single target.

Verbascoside is a natural phenylethanoid glycoside with well-established anti-tumor activity. Previous studies have confirmed the ability of verbascoside to inhibit cell proliferation and induce apoptosis in various cancer cells [7–9]. Whether verbascoside can induce ferroptosis in cancer cells remains unclear; however, in other diseases, such as myocardial ischemia-reperfusion injury, verbascoside regulates ferroptosis [10]. This highlights the potential of verbascoside in modulating apoptosis and ferroptosis. However, whether verbascoside can induce ferroptosis and whether its anti-tumor effects are related to the key node, BCAT2, remain unknown. Based on the critical position of BCAT2 in cancer and its potential for dual regulation of cell death, we propose the core hypothesis that verbascoside, by targeting and inhibiting BCAT2, synergistically triggers both apoptosis and ferroptosis in NSCLC cells. To test this hypothesis, we comprehensively used bioinformatics screening, molecular docking, *in vitro* cellular functional assays, and *in vivo* animal models to systematically elucidate novel targets and mechanisms of verbascoside, with the aim of providing a novel natural product-based therapeutic strategy involving dual death pathways for NSCLC treatment.

## Methods

### Screening of Differentially Expressed Genes (DEGs) in NSCLC

The GSE18842 expression profile dataset was downloaded from the Gene Expression Omnibus database (https://www.ncbi.xyz/gds/), including 46 NSCLC tissue samples and 45 normal lung tissue samples. After data preprocessing and normalization, differential expression analysis was performed using limma package in R. Screening criteria were |logFC| of >0.3 and False Discovery Rate (FDR) of <0.01, ultimately yielding 10,057 DEGs. The primary objective of this step is to perform an intersection analysis of three independent gene sets—differentially expressed genes in NSCLC, predicted VB targets, and ferroptosis-related genes—to identify candidate molecules with functional relevance. This approach aims to identify candidate genes with moderate but biologically significant changes in expression while minimizing false positives.

### Prediction of compound targets

The three-dimensional (3D) structure of verbascoside was downloaded from PubChem (https://pubchem.ncbi.nlm.nih.gov/) and uploaded to PharmMapper (https://www.lilab-ecust.cn/pharmmapper/). A reverse pharmacophore-matching method was used for target prediction. Based on spatial matching scores between pharmacophores and protein-active pockets, the platform-ranked candidate target proteins and highly ranked proteins were selected, yielding 83 potential target proteins.

### Acquisition of ferroptosis-related gene set

A list of ferroptosis-related genes, totaling 484 genes, was downloaded from the FerrDb database (http://www.zhounan.org/ferrdb/current/). This database contains information on literature-verified drivers, suppressors, and markers of ferroptosis.

### Screening of potential target genes and intersection analysis

The potential targets of verbascoside, the NSCLC DEGs, and ferroptosis-related genes were identified. Venn diagrams were used to identify key genes common to all three groups.

### Molecular docking simulation

To verify the interaction between verbascoside and the BCAT2 protein, the 3D structures of verbascoside and BCAT2 were obtained from PubChem and Protein Data Bank databases (https://www.rcsb.org/), respectively. AutoDock Tools (v4.2.6) was used to preprocess the target protein (removing water molecules and ligands, and adding polar hydrogens and Gasteiger charges). Molecular docking was performed using AutoDock Vina1.2.3. Default docking parameters were used, and the Vina score was used to evaluate binding energy, with lower scores indicating a stronger binding affinity.

### Validation of BCAT2 expression using external datasets

The transcriptomic datasets GSE19804 and GSE33532 were downloaded, and BCAT2 expression values were extracted from the standardized matrix data. Differential expression of BCAT2 between tumor and normal samples was assessed using paired Student’s t-tests. Box plots were generated using the ggplot2 R package to visualize differences in expression distributions. Statistical significance was defined as P < 0.05.

### Molecular dynamics simulations

To further evaluate the binding stability and dynamic interactions between Verbascoside and BCAT2, molecular dynamics simulations were performed using GROMACS 2024.5 software in conjunction with the CHARMM36 all-atom force field. Each complex was placed in a cubic box filled with explicit TIP3P water molecules, maintaining a minimum distance of 1.2 nm from the box walls, and neutralized by adding Na⁺ or Cl^-^ ions. Energy minimization was performed using the steepest descent algorithm. Subsequently, the systems were equilibrated for 100 ps under NVT and NPT conditions, respectively, with the temperature maintained at 300 K using a v-rescale thermostat and the pressure maintained at 1 bar using a Parrinello-Rahman constant-pressure thermostat. Finally, a 100 ns production molecular dynamics simulation was conducted under the same conditions. Trajectory analysis was performed using GROMACS’ built-in tools, including root-mean-square deviation (RMSD), root-mean-square fluctuation (RMSF), radius of gyration (Rg), solvent-accessible surface area (SASA), and the number of hydrogen bonds. A two-dimensional free energy landscape (FEL) was constructed based on RMSD and Rg.

### Cell culture

Normal human lung epithelial cells BEAS-2B (C6106) and human lung adenocarcinoma cells NCI-H1299 (C6274), HCC827 (C6298), A549 (C6053), NCI-H1650 (C6641), NCI-H23 (C6669) were purchased from Beyotime Biotechnology Co., Ltd. Complete RPMI-1640 medium was prepared using 10% fetal bovine serum (C0232, Beyotime Biotechnology Co., Ltd., China) and 1% penicillin-streptomycin solution (C0222, Beyotime Biotechnology Co., Ltd., China). All the cells were cultured in RPMI-1640 medium at 37°C in a humidified incubator with 5% CO_2_. Cells at 80–90% confluence were used for subsequent experiments.

### Cell transfection

Cells in the logarithmic growth phase were trypsinized for 3 min, followed by addition of 2 mL of the medium and centrifugation at 1,200 g for 3 min. The supernatant was discarded, cells were resuspended in medium, and cell density was determined using a cell counter (AMQAF1000, Thermo Fisher Scientific Inc, China). The cells were seeded at a density of 1 × 10^5^ per well in six-well plates and incubated overnight. To construct stable BCAT2 knockdown cells in NCI-H1299, the pLKO.1-TRC-puro vector was used. Short hairpin RNA (shRNA) sequences targeting the BCAT2 gene were cloned into an shRNA expression cassette downstream of a U6 promoter. To construct stable BCAT2 overexpressing cells in A549, the pLV-CMV-MCS-pGK-puro vector was used. The full coding sequence of BCAT2 was cloned into multiple cloning sites downstream of the CMV promoter using EcoRI and BamHI restriction enzyme sites.

Virus supernatant (multiplicity of infection = 10) along with 5 ug/mL of polybrene (C0351, Beyotime Biotechnology Co., Ltd, China) was added to the target cell culture medium (NCI-H1299 or A549). After 24 h of infection, the medium was replaced with complete medium containing 1 μmol/L of puromycin (HY-B1743R, MedChemExpress, China) for continuous selection for 2 weeks, until all uninfected control cells died, thereby obtaining stable polyclonal cell pools with integrated exogenous constructs for subsequent functional validation. Primer sequences are detailed in S1 Table.

### Cell Counting Kit-8 (CCK8) assay

Cells in the logarithmic growth phase were seeded at a density of 5,000 cells/well in 96-well plates and incubated overnight. The cells were treated according to the requirements of each group. After treatment, 10 μL of CCK-8 solution (C0039, Beyotime Biotechnology Co., Ltd, China) was added to each well. The 96-well plate was carefully placed in an incubator for 2 h. After incubation, absorbance was measured at 450 nm using a microplate reader.

### Flow cytometry for apoptosis detection

Approximately 1 × 10^5^ cells in the logarithmic growth phase were collected and transferred to sterile centrifuge tubes, centrifuged at 4°C and 1,000 g for 5 min, and the supernatant discarded. The cells were gently washed twice with pre-cooled phosphate-buffered saline (PBS) and centrifuged, and the supernatant was discarded. The cells were resuspended in 100 μL 1 × binding buffer, and 5 μL Annexin V-fluorescein isothiocyanate (FITC) and 5 μL propidium iodide (PI) staining solution (C1062L, Beyotime Biotechnology Co., Ltd, China) were added, gently mixed, and incubated at room temperature in the dark for 15 min. After incubation, 400 μL of binding buffer was added, gently mixed, and cells were analyzed using a flow cytometer (FACSLyric™, BD, USA) within 1 h to determine the percentage of apoptotic cells. Apoptotic cell ratio = early apoptosis (FITC + /PI-) + late apoptosis (FITC + /PI+).

### Flow cytometry for Reactive Oxygen Specis (ROS) detection

The ROS Assay Kit (CA1410; Solarbio, China) was used. An appropriate number of cells were collected and centrifuged at 1000 × g for 5 min, and the supernatant was discarded. The cells were resuspended in DCFH-DA probe diluted at 1:1,000 (final concentration 10 μmol/L), ensuring even suspension. The tubes were incubated at 37°C in the dark for 30 min. After incubation, the cells were washed thrice with serum-free medium, centrifuged at 1000 × g for 5 min, and the supernatant was discarded. Finally, the cells were resuspended in PBS and immediately analyzed. Excitation/emission wavelengths were set at 488/525 nm to detect the intracellular ROS levels.

### Flow cytometry JC-1 assay for mitochondrial membrane potential

A mitochondrial membrane potential assay kit (M8650; Solarbio, China) was used. An appropriate number of cells were collected and centrifuged at 1000 × g for 5 min, and the supernatant was discarded. JC-1 staining working solution was added and mixed, and the tubes were incubated at 37°C in the dark for 20 min. After incubation, the cells were washed twice with pre-cooled JC-1 staining buffer and centrifuging at 1,000 g for 5 min. Finally, the cells were resuspended in an appropriate amount of pre-cooled JC-1 staining buffer and immediately analyzed using a flow cytometer to measure FL1 (green fluorescence) and FL2 (red fluorescence) channel intensities. Changes in the mitochondrial membrane potential were analyzed based on red/green fluorescence intensity ratio.

### Transmission Electron Microscopy (TEM) for mitochondrial structure observation

An appropriate number of cells were collected, transferred to centrifuge tubes, washed 2–3 times with pre-cooled 0.1 mol/L PBS, and centrifuged at 1000 × g for 5 min each time. The cells were fixed overnight at 4°C with 2.5% glutaraldehyde (G5882; Sigma-Aldrich, USA). The next day, the cells were washed thrice with PBS for 10 min each. The cells were then fixed with 1% osmium tetroxide (75632; Sigma-Aldrich, USA) at room temperature in the dark for 2 h. After fixation, the cells were dehydrated using a graded ethanol series (30%, 50%, 70%, 80%, 90%, 95%, and 100%) for 15–20 min at each step. The cells were then infiltrated and embedded in epoxy resin. After polymerization, ultrathin sections (50 nm) were cut. The sections were double-stained with uranyl acetate and lead citrate and observed under a transmission electron microscope (Talos L120C, Thermo Scientific, China) for changes in mitochondrial morphology and structure.

### Animal experiments (Xenograft Tumor Model)

Twenty-four 4–6-week-old male BALB/c nude mice were purchased from Henan Suke Baisi Biological Technology Co., Ltd. (License: SCXK Yu 2020−0005). All the mice were housed in a specific pathogen-free environment with room temperature maintained at 22 ± 2°C and a 12-h light/dark cycle. The animals had free access to sterilized food and water. All animal procedures complied with relevant ethical guidelines and were approved by the Animal Ethics Committee of the Fourth Hospital of Hebei Medical University (Ethics Approval No. 2025536).

NCI-H1299 cells in the logarithmic growth phase were trypsinized, resuspended in serum-free medium, and counted. A suspension of 100 μL RPMI-1640 medium containing 3 × 10^6^ cells was subcutaneously injected into the axillary region of the mice to establish a xenograft model. After inoculation, the mice were randomly divided into four groups (six mice per group) using a random number table. The general condition and tumor growth of the mice were monitored regularly. Tumor length (a) and width (b) were measured weekly using calipers, and tumor volume was calculated using the formula V = 1/2 × a × b² to record tumor growth curves. Groups requiring verbascoside intervention received intraperitoneal injections at a dose of 40 mg/kg [11], starting on the day of inoculation (day 0) and then every 3 days until day 33. The study endpoint was set at day 35. All tumor-bearing mice were euthanized by CO_2_ inhalation, and death was confirmed by cervical dislocation. Excised tumor tissues were collected for subsequent molecular biology analyses. During the experiment, all mice were observed at least twice a day to monitor their overall health and signs related to humane endpoints. The pre-defined humane endpoints in this study were as follows: tumor volume exceeding 2000 mm^3^, tumor ulceration or infection, weight loss exceeding 20% of the initial body weight, persistent lethargy, hunched back, unkempt fur, loss of appetite, difficulty in breathing, significant decrease in activity, or signs of severe pain and distress. If a mouse reached any of these endpoints, it was immediately euthanized by CO_2_ inhalation combined with cervical dislocation to prevent unnecessary suffering. All animal conditions, intervention measures, and euthanasia procedures were recorded in detail. In this study, animal death was not set as a direct experimental endpoint. All animals were euthanized either at the pre-determined time points or when signs of humane endpoints emerged, which complies with the requirements of laboratory animal welfare and ethics. In this study, none of the mice showed signs of humane endpoints before day 35.

### Enzyme-linked immunosorbent assay

The reduced glutathione (GSH) and oxidized glutathione disulfide (GSSG) (A061-1, NanJing JianCheng Bioengineering Institute, China), malondialdehyde (MDA) (S0131M, Beyotime Biotechnology, China), and Fe²⁺ (BC5415, Solarbio, China) detection kits were used. Prior to the experiment, all kit components were equilibrated at room temperature. Standards were serially diluted according to the manufacturer’s instructions and added to the standard wells of the microplate, and prepared samples were added to the sample wells. When the color development was appropriate, a stop solution was added to terminate the reaction. Absorbance was immediately measured using a microplate reader: GSH/GSSG was measured at 405 nm, MDA was at 532 nm, and Fe² ⁺ was at 593 nm. Amounts of GSH/GSSG, MDA, and Fe²⁺ in the samples was calculated based on the formulas provided in the instructions.

### Reverse Transcriptase Quantitative Polymerase Chain Reaction (RT-qPCR)

Cells from each group were collected, and 1 mL of TRIzol reagent was added, followed by incubation at room temperature for 5 min. Approximately 0.2 mL of chloroform was then added, the mixture was vigorously shaken for 15 s, and left at room temperature for 3 min. The mixture was centrifuged at 4°C and 12,000 g for 15 min, and the upper aqueous phase was transferred to a new tube. An equal volume of isopropanol was then added, mixed, and incubated at room temperature for 10 min. After centrifugation at 4°C and 12,000 g for 10 min, the supernatant was discarded. The RNA pellet was washed twice with 75% ethanol, centrifuged at 4°C and 7,500 g for 5 min each time, and the supernatant was discarded. The RNA pellet was air-dried at room temperature and dissolved in 50 μL DEPC-treated water. For cDNA synthesis, 1 μg RNA was reverse transcribed using PrimeScript™ RT Reagent Kit (RR047A, Takara, China) following the manufacturer’s instructions. qPCR amplification was performed using the synthesized cDNA as a template with TB Green® Premix Ex Taq™ II (RR820A; Takara, China). The reaction conditions were: 95°C for 30 s pre-denaturation, followed by 40 cycles of 95°C for 5 s and 60°C for 30 s. The relative expression of the target gene was determined by comparing its cycle threshold value with that of the internal reference gene, and expressed as fold change relative to a control group using the 2^(-ΔΔCT) method [14]. Primer sequences: BCAT2 forward, 5’-AATTATGGGCCCACCGTGTT-3’; BCAT2 reverse, 5’-TGTTCATGGTTCCCACCTCG-3.’ glyceraldehyde-3-phosphate dehydrogenase (GAPDH) forward, 5’-GTCAAGGCTGAGAACGGGAA-3’; GAPDH reverse, 5’-AAATGAGCCCCAGCCTTCTC-3.’ Other primer sequences can be found in S1 Table.

### Western blot

Cells were placed on ice, the medium was aspirated, and the cells were washed three times with pre-cooled PBS. Approximately 500 μL RIPA lysis buffer (P0013B, Beyotime Biotechnology, China) was then added, and the cells were lysed on ice for 30 min. The lysate was transferred to a centrifuge tube, centrifuged at 4°C and 12,000 g for 15 min, and the supernatant was collected. Protein concentration was determined using a BCA Protein Assay Kit (P0012, Beyotime Biotechnology, China). Equal amounts of protein were mixed with the loading buffer and denatured by boiling for 5 min. Protein samples were loaded onto pre-cast gels (4569034; Bio-Rad, China) for sodium dodecyl sulfate-polyacrylamide gel electrophoresis electrophoresis (120 V, 70 min). After the electrophoresis, the proteins were transferred onto polyvinylidene difluoride membranes (240 mA for 60 min). The membranes were blocked with 5% skim milk at room temperature for 2 h. After the blocking, the membranes were incubated with corresponding primary antibodies overnight at 4°C. The next day, the membranes were washed thrice with Tris-buffered saline with 0.1% Tween® 20 (TBST) for 10 min each and then incubated with the corresponding secondary antibodies at room temperature for 2 h. The membranes were washed thrice with TBST for 10 min each. Finally, the membranes were incubated with an enhanced chemiluminescent substrate (32106, Thermo Fisher Scientific, China) and imaged. Relative protein expression was calculated as a grayscale value of the target protein divided by that of GAPDH. Detailed antibody information is shown in S2 Table.

### Statistical analysis

All experiments were biologically replicated at least three times to ensure the reliability and reproducibility of the results.. Data are presented as mean ± standard deviation. Statistical analyses were performed using GraphPad Prism software. An unpaired two-tailed t-test was used for comparisons between two groups. A one-way analysis of variance was used to compare multiple groups.

## Results

### Screening of Ferroptosis-related genes affected by verbascoside in NSCLC

**Fig 1:**
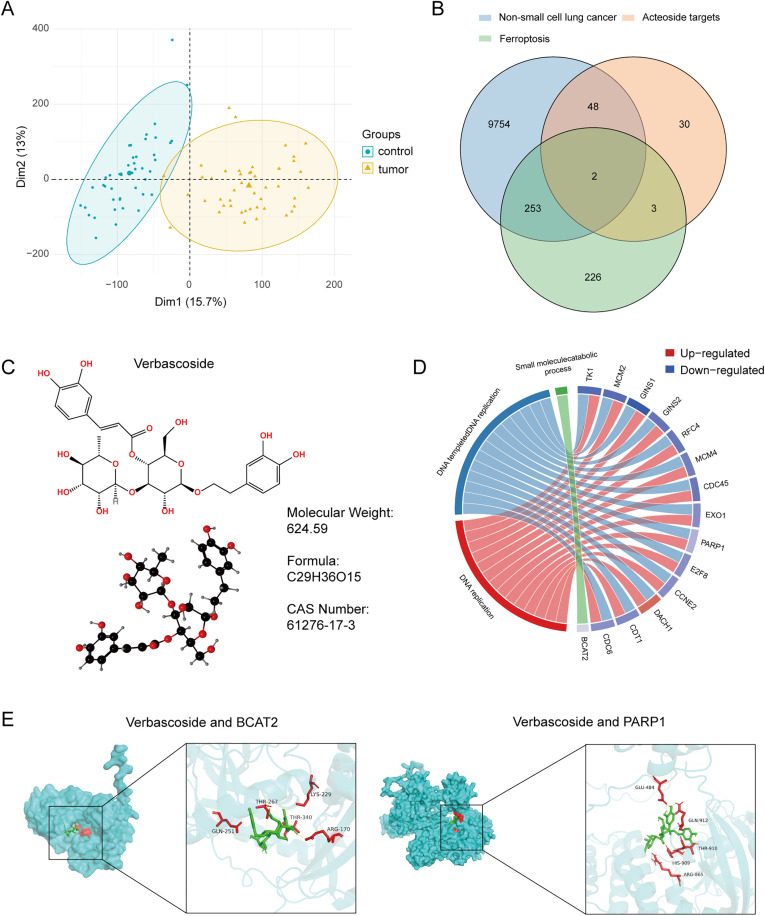
Screening of ferroptosis-related genes affected by verbascoside in NSCLC. The GSE18842 expression profile dataset was downloaded from the GEO database, and principal component analysis (PCA) was performed on the GSE18842 dataset. (B) Venn diagram showing the overlapping genes among three sets: differentially expressed genes in NSCLC, predicted targets of verbascoside, and ferroptosis-related genes. (C) Information on the two- and three-dimensional structural formulas, relative molecular weight, and CAS number of verbascoside. (D) Circos plot of Gene Ontology (GO) biological process enrichment, presenting the enriched functional pathways of the overlapping genes and the expression status of corresponding genes. (E) Molecular docking of verbascoside with BCAT2 and PARP1 proteins.

Principal component analysis of the GSE18842 dataset showed a slight overlap between control and tumor groups, with majority of samples showing clear separation, indicating significant transcriptomic differences between the two groups (Fig 1A). Subsequently, differential expression analysis was performed using R limma package, with criteria set at |logFC| > 0.3 and FDR < 0.01, ultimately identifying 10,057 DEGs. A total of 484 ferroptosis-related genes were obtained from the FerrDb database; simultaneously, 83 potential drug target proteins with high-ranking scores were screened via pharmacophore matching and active site scoring on the PharmMapper platform. Considering the intersection of these three sets using a Venn diagram, two key overlapping genes were identified: BCAT2 and poly (ADP-ribose) polymerase 1 (PARP1) (Fig 1B).

Fig 1C displays the two-dimensional and 3D chemical structures of verbascoside, along with its relative molecular mass and CAS number. Fig 1D shows the pathway enrichment of the genes; BCAT2 is associated with the small molecule catabolic process, while PARP1 is associated with pathways related to DNA replication. Molecular docking simulations were performed to evaluate the binding potential of verbascoside to the two target proteins. Results showed that the binding energy between verbascoside and PARP1 was −4.42 kcal/mol, indicating a moderate binding affinity; whereas the binding energy with BCAT2 was −7.8 kcal/mol, suggesting a stronger binding capability (Fig 1E). Based on the docking results, BCAT2, with its strong binding ability, was selected as the primary target for subsequent in-depth analysis.

**Fig 2:**
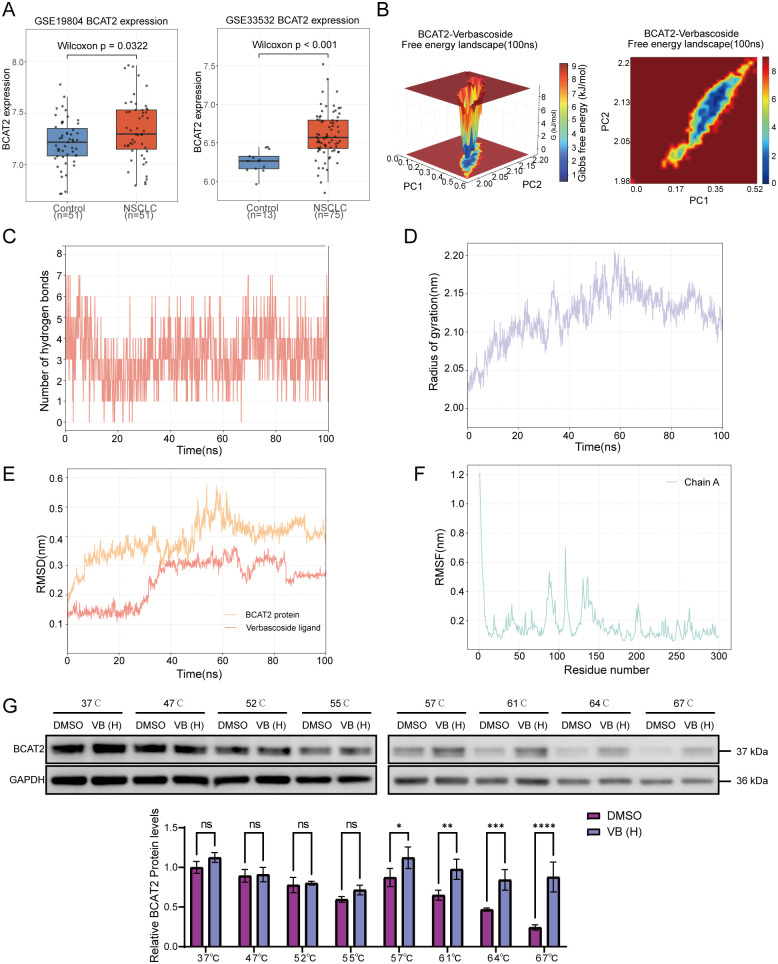
Verification of the direct binding between verbascoside and BCAT2. (A) Box plots showing BCAT2 mRNA expression levels in normal lung tissues and NSCLC tissues from the GSE19804 and GSE33532 datasets. (B) Free energy landscape (FEL) of the BCAT2-verbascoside complex during 100 ns molecular dynamics simulation, mapped along the first two principal components (PC1 and PC2). (C) Time evolution of the number of hydrogen bonds formed between verbascoside and BCAT2 protein throughout the 100 ns simulation trajectory. (D) Time course of the radius of gyration (Rg) of BCAT2 protein during the simulation, reflecting the overall compactness of the protein tertiary structure. (E) Root mean square deviation (RMSD) trajectories of the BCAT2 protein backbone and verbascoside ligand over the 100 ns molecular dynamics simulation. (F) Root mean square fluctuation (RMSF) profile of BCAT2 amino acid residues, showing the flexibility of each residue upon verbascoside binding. (G) Cellular thermal shift assay (CETSA) results. Representative Western blot bands of BCAT2 protein in the DMSO group and high-dose verbascoside (VB (H)) group across a temperature gradient, with GAPDH as the loading control (n = 3). *p < 0.05; **p < 0.01; ***p < 0.001; ****p < 0.0001, ns: No significant difference.

To further validate the expression pattern of BCAT2 in NSCLC, we retrieved two large-scale independent GEO datasets, GSE19804 and GSE33532. BCAT2 exhibited markedly higher expression levels in NSCLC tumor tissues compared to normal lung tissues in both external validation datasets, confirming that BCAT2 is transcriptionally upregulated in NSCLC (Fig 2A). We performed a 100 ns molecular dynamics simulation based on the optimal docking conformation. The free energy landscape was applied to characterize the conformational energy distribution of the binding system (Fig 2B). The BCAT2-verbascoside complex converged to a well-defined low-energy basin, indicating that this binding conformation is thermodynamically stable and energetically favorable. Throughout the simulation trajectory, the number of hydrogen bonds formed between verbascoside and BCAT2 fluctuated within a stable range (Fig 2C). The Rg of BCAT2 increased gradually and then plateaued, suggesting that the overall compactness and tertiary structure of BCAT2 were well preserved after ligand binding (Fig 2D). RMSD analysis revealed that both the BCAT2 protein backbone and the verbascoside ligand reached conformational equilibrium after approximately 40 ns, and their RMSD values fluctuated within a narrow range for the remainder of the simulation (Fig 2E). Root mean square fluctuation (RMSF) analysis demonstrated that most amino acid residues of BCAT2 exhibited low flexibility, with only a few loop regions showing moderate fluctuations (Fig 2F), which further verified the structural stability of BCAT2 upon verbascoside binding.

### CETSA validates the direct binding of verbascoside to BCAT2 in cells

To confirm the direct interaction between verbascoside and BCAT2 in a physiological cellular environment, we conducted the cellular thermal shift assay (CETSA). As shown in Fig 2G, BCAT2 protein levels in both groups declined progressively with rising temperature due to heat-induced denaturation. Notably, at temperatures of 57°C and above, the residual BCAT2 protein level in the high-dose verbascoside (VB (H)) group was significantly higher than that in the DMSO control group, with statistical significance observed across the 57°C to 67°C temperature range. This thermal stabilization effect demonstrates that verbascoside physically binds to endogenous BCAT2 protein in NCI-H1299 cells, thereby protecting the protein from thermal denaturation.

### BCAT2 induces apoptosis and ferroptosis in NSCLC cells

To investigate the expression pattern of BCAT2 in NSCLC, we examined its mRNA and protein expression levels in human normal lung epithelial cells (BEAS-2B) and five NSCLC cell lines (NCI-H1299, HCC827, A549, NCI-H1650, and NCI-H23). Results showed that compared with BEAS-2B cells, BCAT2 expression was upregulated to varying degrees in all the tested NSCLC cell lines. Among them, NCI-H1299 cells showed the most significant upregulation of BCAT2 mRNA and protein, whereas the upregulation in A549 cells was relatively weaker (Fig 2A and 2B). Based on this expression profile, to explore the effects of BCAT2 loss-of-function and gain-of-function on cell fate, we established stable BCAT2-knockdown cell lines in NCI-H1299 cells with high BCAT2 expression, and stable BCAT2-overexpressing cell lines in A549 cells with low BCAT2 expression. Western blotting and qPCR confirmed the efficiency of BCAT2 knockdown and overexpression in the respective cell models (S1 Fig).

**Fig 3:**
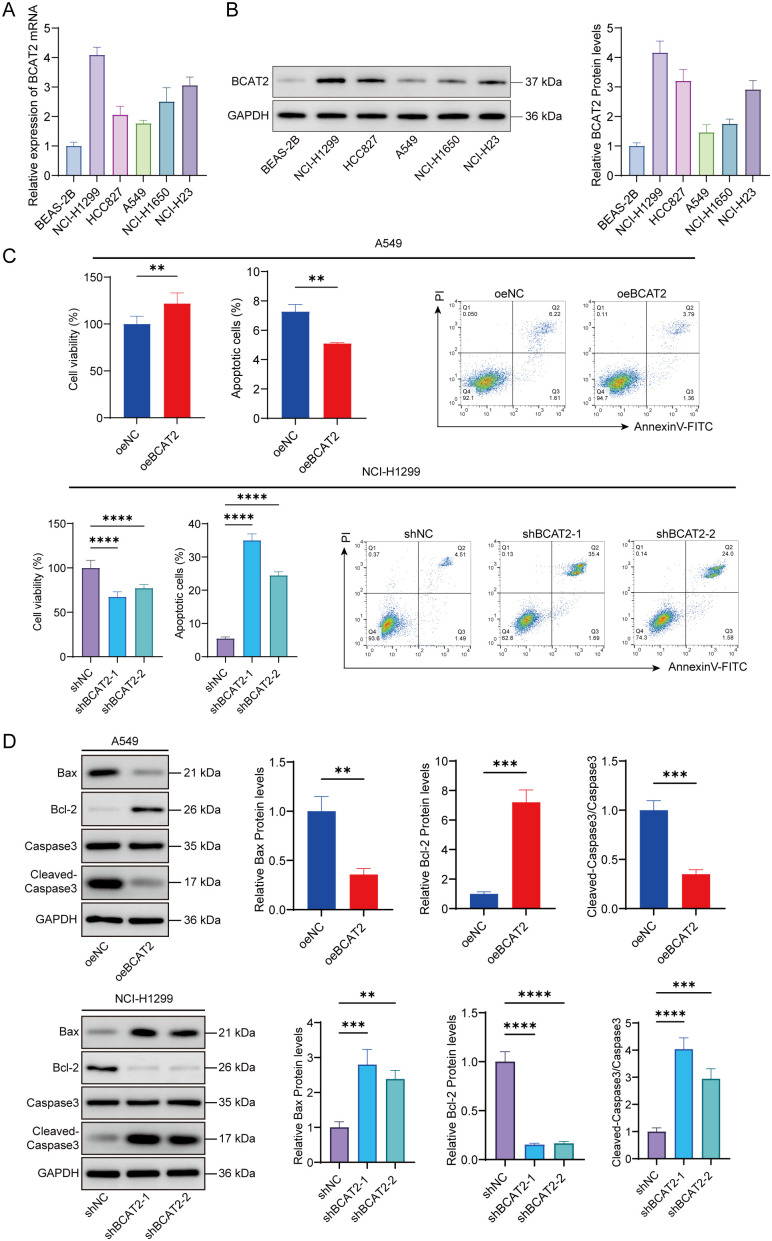
BCAT2 modulates cell viability and apoptosis in NSCLC cells. (A) Relative expression levels of BCAT2 mRNA in BEAS-2B, NCI-H1299, HCC827, A549, NCI-H1650, and NCI-H23 cells were detected by RT-qPCR (n = 3). (B) Relative expression levels of BCAT2 protein in BEAS-2B, NCI-H1299, HCC827, A549, NCI-H1650, and NCI-H23 cells were detected by Western blot (n = 3). (C) Effects of BCAT2 overexpression (in A549 cells, upper panel) and BCAT2 knockdown (in NCI-H1299 cells, lower panel) on cell viability and apoptosis (n = 3). (D) Western blot analysis of apoptosis-related proteins (Bax, Bcl-2, Caspase-3, cleaved Caspase-3) in BCAT2-overexpressing A549 cells (upper panel) and BCAT2-knockdown NCI-H1299 cells (lower panel), with corresponding quantitative analysis on the right. GAPDH served as the loading control (n = 3). **p < 0.01; ***p < 0.001; ****p < 0.0001.

CCK-8 assays showed that BCAT2 overexpression significantly enhanced the viability of A549 cells, whereas BCAT2 knockdown significantly reduced the viability of NCI-H1299 cells (Fig 3C). Flow cytometry analysis of apoptosis revealed that BCAT2 overexpression decreased the apoptotic rate in A549 cells, accompanied by downregulation of the pro-apoptotic protein Bax, upregulation of the anti-apoptotic protein Bcl-2, and a decrease in the cleaved-caspase-3/caspase-3 ratio. Conversely, knockdown of BCAT2 significantly increased the apoptotic rate in NCI-H1299 cells and induced Bax upregulation, Bcl-2 downregulation, and an increase in the cleaved-caspase-3/caspase-3 ratio (Fig 3C and 3D). Collectively, these results indicate that BCAT2 knockdown can induce apoptosis in NSCLC cells by regulating the expression of apoptosis-related proteins.

**Fig 4:**
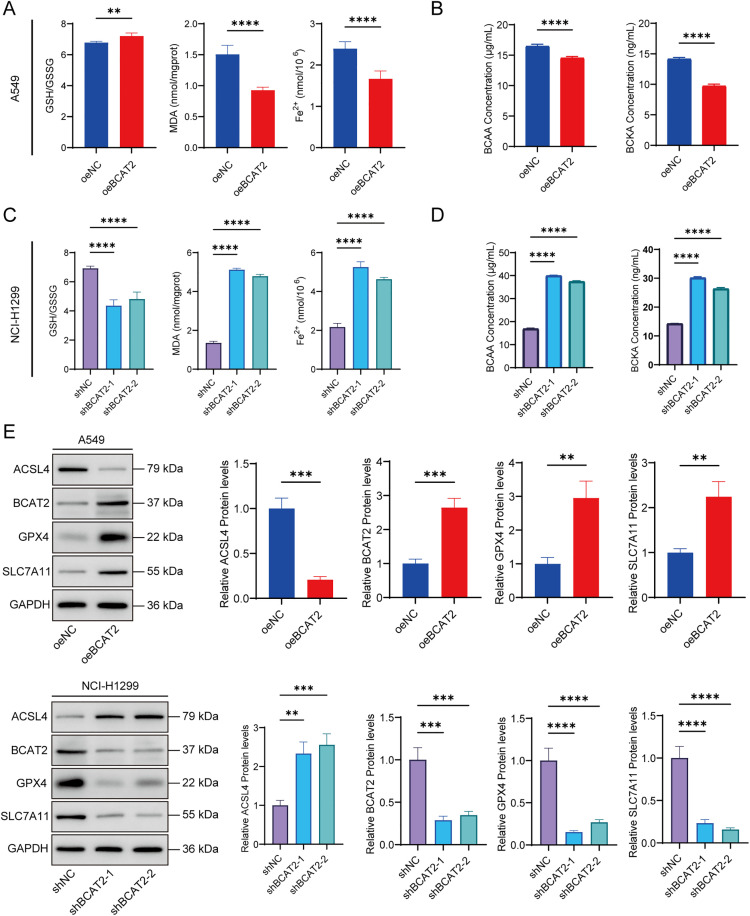
BCAT2 regulates ferroptosis and branched-chain amino acid metabolism in NSCLC cells. (A) Detection of ferroptosis-related biochemical indicators in BCAT2-overexpressing A549 cells, including the GSH/GSSG ratio, malondialdehyde (MDA) level and intracellular Fe² ⁺ content (n = 3). (B) Intracellular concentrations of branched-chain amino acids (BCAA) and branched-chain α-keto acids (BCKA) in BCAT2-overexpressing A549 cells (n = 3). (C) Detection of the GSH/GSSG ratio, MDA level and Fe² ⁺ content in BCAT2-knockdown NCI-H1299 cells (n = 3). (D) Intracellular concentrations of BCAA and BCKA in BCAT2-knockdown NCI-H1299 cells (n = 3). (E) Western blot analysis of ferroptosis-related proteins (ACSL4, GPX4, SLC7A11) and BCAT2 in BCAT2-overexpressing A549 cells (upper panel) and BCAT2-knockdown NCI-H1299 cells (lower panel), with corresponding quantitative analysis on the right. GAPDH served as the loading control (n = 3). **p < 0.01; ***p < 0.001; ****p < 0.0001.

After confirming that BCAT2 knockdown effectively induced apoptosis, we further explored the effects of BCAT2 on ferroptosis. BCAT2 overexpression in A549 cells increased intracellular GSH/GSSG ratio, while significantly decreasing MDA and Fe² ⁺ levels (Fig 4A). simultaneously, the downstream substrates of BCAT2, branched-chain amino acids (BCAA) and branched-chain keto acids (BCKA), also showed significant decreases (Fig 4B). This is because the catabolic metabolism of BCAA within the cells is regulated by BCAT2, and BCAA can be converted into BCKA [12,13]. The ferroptosis positive regulator ACSL4 expression decreased, and the negative regulators GPX4 and SLC7A11 expression increased (Fig 4E). In contrast, knockdown of BCAT2 in A549 cells led to a decreased GSH/GSSG ratio, accumulation of MDA, Fe² ⁺ , BCAA and BCKA, upregulation of ACSL4 protein, and downregulation of GPX4 and SLC7A11 protein (Fig 4C, 4D and 4E). These results indicate that BCAT2 knockdown induces both apoptosis and ferroptosis in NSCLC cells.

### Effects of BCAT2 on ferroptosis-related proteins reversed by erastin and ferrostatin-1

To further validate the regulatory role of BCAT2 in ferroptosis of NSCLC cells, we performed rescue experiments with the ferroptosis inducer erastin and the ferroptosis inhibitor Fer-1 in BCAT2-overexpressing and BCAT2-knockdown cell models, respectively. As shown in S2 Fig. BCAT2 overexpression in A549 cells exerted an inhibitory effect on ferroptosis. When combined with erastin treatment, the alterations of ferroptosis-related proteins were significantly reversed, manifested as upregulated ACSL4 expression and downregulated GPX4 and SLC7A11 expression. In NCI-H1299 cells, BCAT2 knockdown induced a ferroptosis-promoting phenotype, and the addition of Fer-1 effectively counteracted the changes in ferroptosis-related proteins. These findings indicate that BCAT2 functions as a negative regulator of ferroptosis in NSCLC cells by modulating key molecules in the ferroptosis pathway.

### BCAT2 induces mitochondrial damage in NSCLC cells

**Fig 5:**
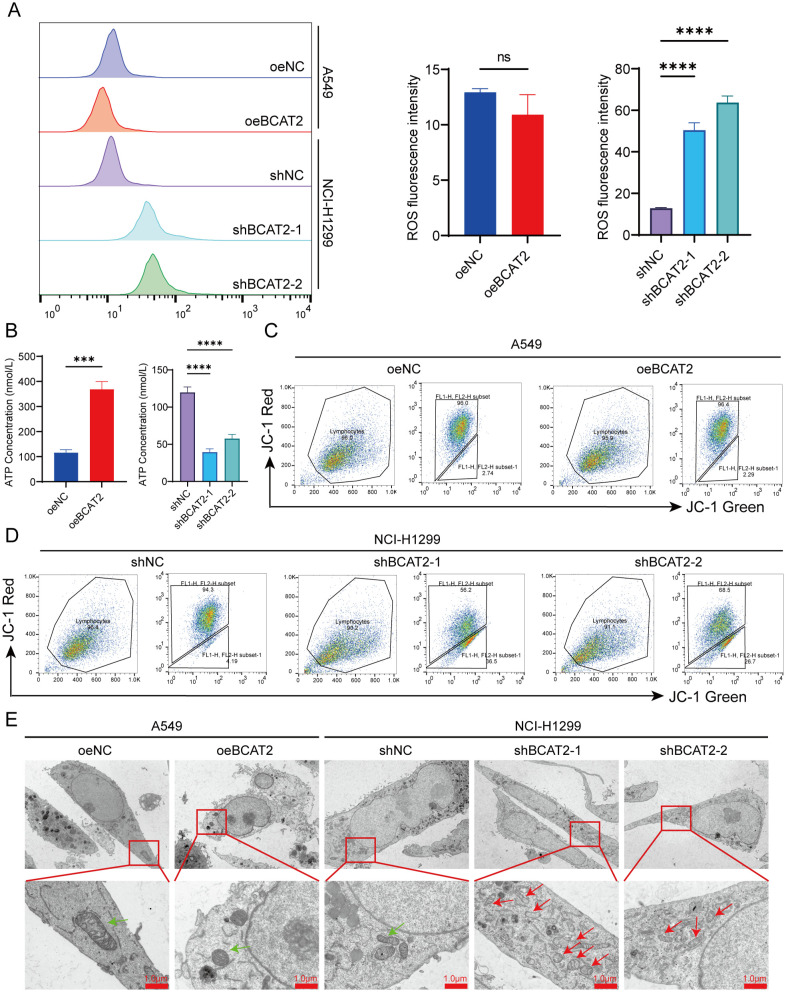
BCAT2 regulates mitochondrial function and ultrastructure in NSCLC cells. (A) Changes in ROS levels in A549 and NCI-H1299 cells were detected by flow cytometry (n = 3). (B) Changes in ROS levels in H1299 cells were detected by flow cytometry (n = 3). (C) Changes in mitochondrial membrane potential in different groups of A549 cells were detected by flow cytometry (JC-1) (n = 3). (D) Changes in mitochondrial membrane potential in different groups of NCI-H1299 cells were detected by flow cytometry (JC-1) (n = 3). (E) Mitochondrial morphology and function in A549 and H1299 cells were observed by transmission electron microscopy (10,000 × , scale bar: 1 μm) (n = 3). ***p < 0.001; ****p < 0.0001, ns: No significant difference.

To further investigate the potential mechanism by which BCAT2 knockdown induces NSCLC cell death, we examined changes in mitochondrial function and morphology. Results showed that intracellular ROS levels did not change significantly in BCAT2-overexpressing cells, whereas BCAT2 knockdown caused a significant accumulation of ROS (Fig 5A). We also observed ATP generation; BCAT2 overexpression contributed to ATP generation, whereas knockdown of BCAT2 inhibited it (Fig 5B). JC-1 staining showed no significant change in mitochondrial membrane potential after BCAT2 overexpression, whereas BCAT2 knockdown led to a decrease in the mitochondrial membrane potential (Fig 5C and 5D). TEM further confirmed that BCAT2 overexpression helped maintain mitochondrial structural integrity (green arrows indicate normal mitochondria), whereas BCAT2 knockdown induced typical damage morphology such as mitochondrial swelling and cristae rupture (red arrows indicate damaged mitochondria) (Fig 5E). These results suggest that BCAT2 regulates the death of NSCLC cells by affecting mitochondrial function and structural integrity.

### Verbascoside induces apoptosis and ferroptosis in NSCLC cells by inhibiting BCAT2

In previous studies, we confirmed that BCAT2 knockdown effectively induces apoptosis and ferroptosis in NSCLC cells. Based on molecular docking experiments suggesting a binding site between verbascoside and BCAT2, we investigated whether verbascoside could target and inhibit BCAT2 expression. We selected the NSCLC cell line, NCI-H1299, which endogenously expresses high BCAT2 levels, to validate the direct targeting effect of verbascoside on BCAT2.

**Fig 6:**
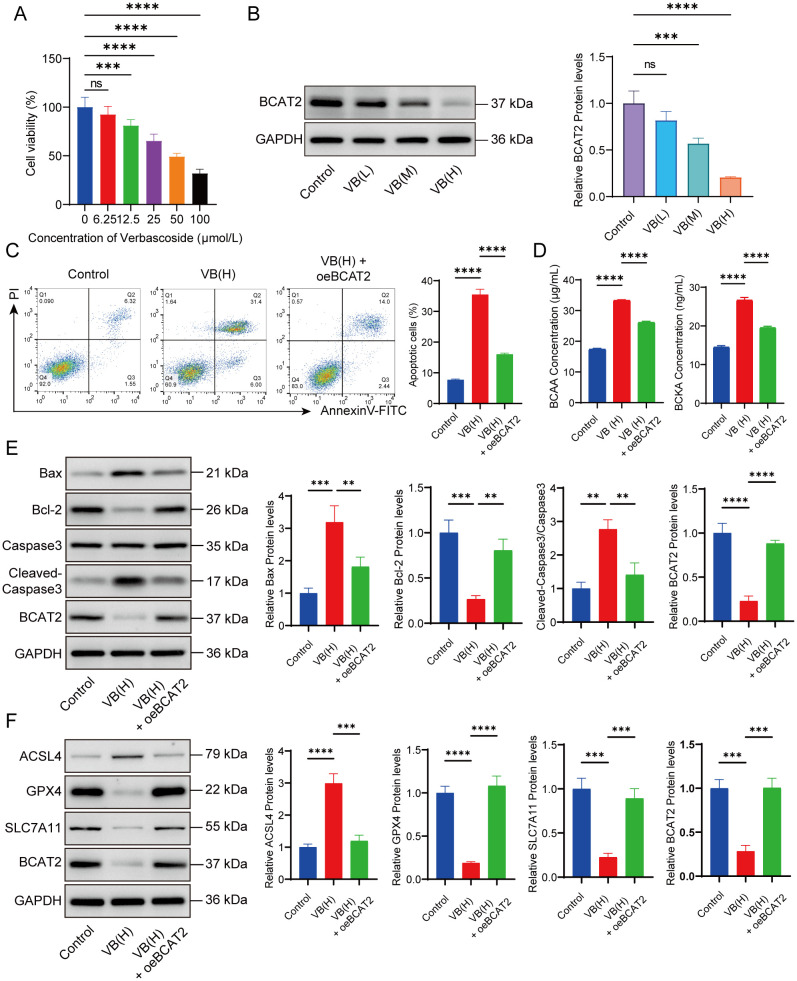
Verbascoside induces apoptosis and ferroptosis in NCI-H1299 cells by inhibiting BCAT2. (A) CCK-8 assay showing the dose-dependent inhibitory effect of VB (0, 6.25, 12.5, 25, 50, 100 μmol/L) on the viability of NCI-H1299 cells (n = 3). (B) After treatment with different concentrations of VB in NCI-H1299 cells, changes in BCAT2 protein expression were detected by Western blot (n = 3). (C) Changes in apoptosis in NCI-H1299 cells were detected by flow cytometry using FITC/PI double staining (n = 3). (D) Relative expression of Bax, Bcl-2, caspase-3, and cleaved-caspase3 proteins in NCI-H1299 cells after different treatments was detected by Western blot (n = 3). (E) Relative expression of ACSL4, GPX4, and SLC7A11 proteins in NCI-H1299 cells after different treatments was detected by Western blot (n = 3). **p < 0.01; ***p < 0.001; ****p < 0.0001, ns: No significant difference.

First, treatment of NCI-H1299 cells with different concentrations of verbascoside (6.25, 12.5, 25, 50, and 100 μmol/L) showed that verbascoside inhibited cell viability in a concentration-dependent manner, with the inhibitory effect on cell proliferation significantly increasing with higher drug concentrations (Fig 6A). Furthermore, treatment with 25 (VB(L)), 50 (VB(M)), and 100 μmol/L verbascoside(VB(H)) for 48 h showed significant inhibition of BCAT2 expression by Western blot, with the 100 μmol/L treatment group (VB(H)) showing the most pronounced effect; therefore, this concentration was used for subsequent experiments (Fig 6B). We simultaneously measured the protein levels of PARP1 and γ-H2AX, a classic marker of DNA damage. The ratio of cleaved PARP1 to total PARP1 showed no significant decrease in the VB(L) and VB(M) groups; only the VB(H) group exhibited a statistically significant reduction, yet the magnitude of this downregulation was far smaller than that of BCAT2 (S3 Fig). Furthermore, the protein level of γ-H2AX, a canonical biomarker of DNA double-strand breaks, remained largely unchanged across all VB treatment groups, indicating that the anti-tumor effect of verbascoside is not exerted by inhibiting PARP1 and blocking DNA repair.

Flow cytometry analysis revealed that treatment with VB(H) significantly increased the apoptotic rate compared to the control (Fig 6C). VB treatment reduces BCAT2 levels, leading to a significant accumulation of BCAA and BCKA, whereas overexpression of BCAT2 reduces the accumulation of BCAA and BCKA (Fig 6D). Western blot analysis showed increased expression of the pro-apoptotic protein, Bax, decreased BCAT2 expression and the anti-apoptotic protein, Bcl-2, and an increased cleaved-caspase-3/caspase-3 ratio. BCAT2 overexpression can reverse these apoptosis-related changes induced by verbascoside, indicating that verbascoside induces apoptosis by targeting and inhibiting BCAT2 (Fig 6E).

Additionally, we examined the expression of ferroptosis markers. After VB(H) treatment, ACSL4 expression significantly increased, whereas GPX4, SLC7A11, and BCAT2 expression significantly decreased. Similarly, BCAT2 overexpression counteracted the regulatory effects of VB(H) on ferroptosis-related proteins (Fig 6F). These results indicated that verbascoside induces apoptosis and ferroptosis in NSCLC cells by targeting and inhibiting BCAT2 expression.

### Verbascoside induces mitochondrial damage in NSCLC cells by inhibiting BCAT2

**Fig 7:**
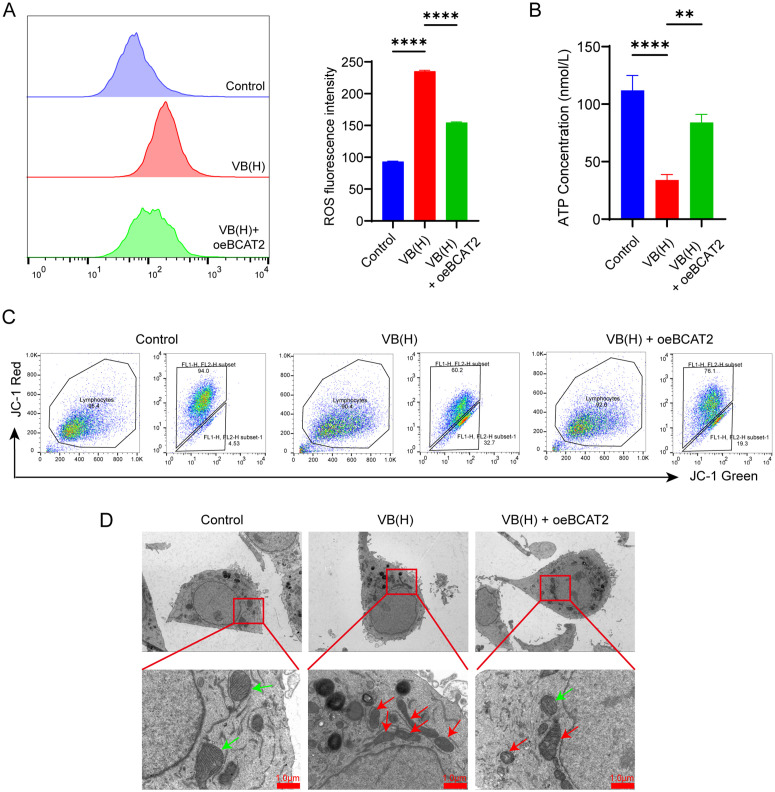
Verbascoside Induces Mitochondrial Damage in NCI-H1299 cells by Inhibiting BCAT2. (A) Changes in ROS levels in different groups of NCI-H1299 cells were detected by flow cytometry (n = 3). (B) Intracellular ATP concentrations in the NCI-H1299 cells (n = 3). (C) Changes in mitochondrial membrane potential in different groups of NCI-H1299 cells were detected by flow cytometry (JC-1) (n = 3). (D) Mitochondrial morphology and function in NCI-H1299 cells were observed by transmission electron microscopy (10,000 × , scale bar: 1 μm) (n = 3). **p < 0.01; ****p < 0.0001.

Results showed that VB(H) significantly increased intracellular ROS levels, whereas BCAT2 overexpression effectively suppressed ROS accumulation (Fig 7A). In terms of energy metabolism, VB(H) significantly reduced ATP generation, an effect that was reversed by BCAT2 overexpression (Fig 7B). JC-1 staining results indicated a significant decrease in mitochondrial membrane potential in the VB(H) group, whereas BCAT2 overexpression helped maintain membrane potential stability (Fig 7C). TEM further confirmed at the ultrastructural level that VB(H) induced typical mitochondrial damage, such as swelling and cristae rupture (indicated by red arrows), whereas BCAT2 overexpression maintained mitochondrial structural integrity (indicated by green arrows) (Fig 7D). These results indicate that verbascoside disrupts mitochondrial function and structure by targeting and inhibiting BCAT2, thereby participating in the regulation of NSCLC cell death.

### Verbascoside induces ferroptosis and apoptosis in tumor tissues of xenograft mouse models by inhibiting BCAT2

**Fig 8:**
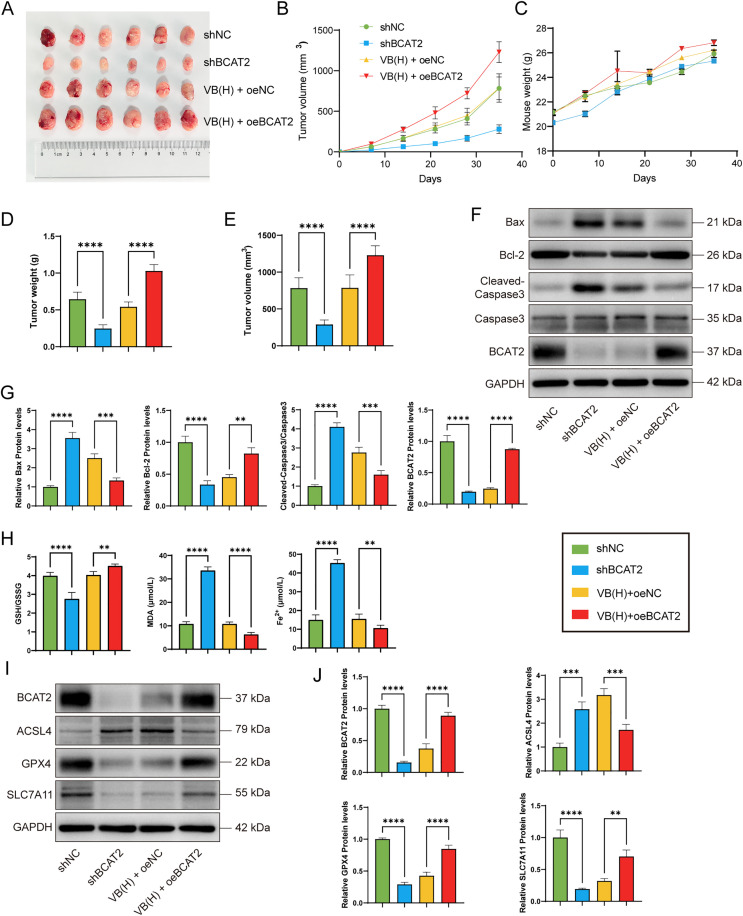
Verbascoside inhibits NSCLC xenograft tumor growth and induces apoptosis and ferroptosis in vivo via targeting BCAT2. (A) Representative macroscopic photographs of excised tumor tissues (n = 6). (B) Tumor volume growth curves of each group over the entire experimental period. (n = 6). (C) Body weight curves of mice in each group during the experiment (n = 6). (D) Statistical graph of tumor weight in mice after dissection (n = 6). (E) Statistical graph of tumor volume in mice after dissection (n = 6). (F) Western blot detection of apoptosis-related proteins (Bax, Bcl-2, cleaved Caspase-3, Caspase-3, and BCAT2) in tumor tissues of each group, with GAPDH as the loading control (n = 3). (G) Quantitative analysis of the relative protein levels corresponding to Fig F. (n = 3). (H) Detection of ferroptosis-related biochemical indicators in tumor tissues, including the GSH/GSSG ratio, MDA content, and Fe² ⁺ level (n = 6). (I) Western blot detection of ferroptosis-related proteins (BCAT2, ACSL4, GPX4, and SLC7A11) in tumor tissues of each group, with GAPDH as the loading control (n = 3). (J) Quantitative analysis of the relative protein levels corresponding to Fig I (n = 3). **p < 0.01; ***p < 0.001; ****p < 0.0001.

In previous *in vitro* studies, we confirmed that verbascoside induces apoptosis and ferroptosis in NSCLC cells by targeting BCAT2. To further validate this effect *in vivo*, we established a nude mouse xenograft tumor model using NCI-H1299 cells. Experimental results showed that compared with the control group, either direct knockdown of BCAT2 or intraperitoneal injection of verbascoside significantly inhibited tumor volume growth and weight increase, however, there was no significant change in the weight of the mice. (Fig 8A-8E). BCAT2 overexpression along with verbascoside treatment partially restored tumor growth, suggesting a key role of BCAT2 in tumor growth inhibition by verbascoside.

**Fig 9:**
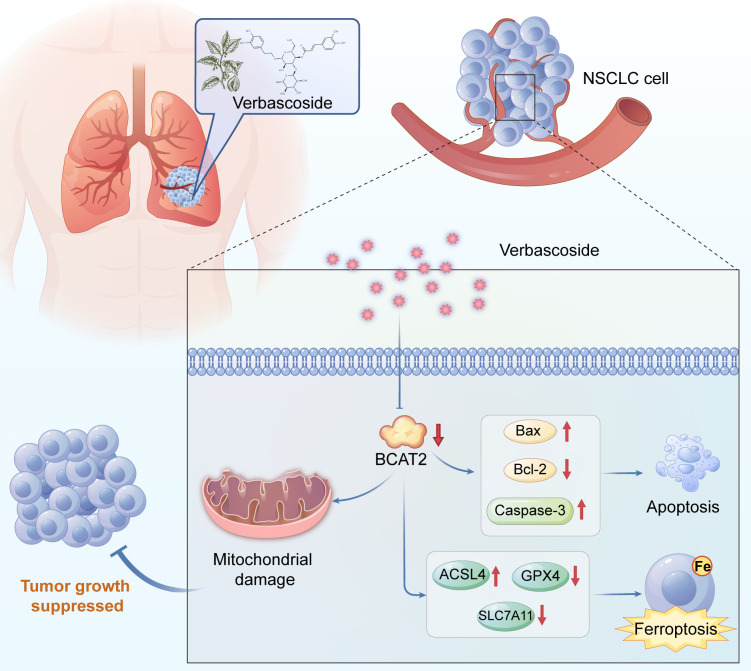
Schematic illustration of the antitumor mechanism of verbascoside against non-small cell lung cancer via targeting BCAT2.

To investigate whether verbascoside induces ferroptosis and apoptosis in tumor tissues *in vivo*, we examined the expression of related markers. Results showed that BCAT2 knockdown or verbascoside treatment significantly upregulated the pro-apoptotic protein, Bax, downregulated the anti-apoptotic proteins, Bcl-2 and BCAT2, and increased the cleaved-caspase-3/caspase-3 ratio. In contrast, BCAT2 overexpression during verbascoside treatment reversed these apoptosis-related changes (Fig 8F and 8G). Furthermore, regarding ferroptosis-related indicators, BCAT2 knockdown or verbascoside treatment led to a significant decrease in the GSH/GSSG ratio, increased MDA and Fe² ⁺ content, upregulated ACSL4 expression, and significantly decreased GPX4, SLC7A11, and BCAT2 expression. Similarly, BCAT2 overexpression antagonized the regulatory effects of verbascoside on these ferroptosis markers (Fig 8H and 8I). Collectively, these *in vivo* data demonstrate that verbascoside can effectively induce apoptosis and ferroptosis in NSCLC cells by targeting and inhibiting BCAT2. Fig 9 illustrates the specific mechanism of this study. Verbascoside targets and inhibits the core node BCAT2, simultaneously activating two programmed death pathways, apoptosis and ferroptosis, causing mitochondrial dysfunction in NSCLC.

## Discussion

Non-small cell lung cancer (NSCLC) remains a leading cause of cancer-related deaths worldwide, and the development of safe and effective therapeutic agents targeting metabolic vulnerabilities in tumors represents a promising direction in anticancer drug research and development. In this study, we integrated transcriptomic differential analysis, drug target prediction, and ferroptosis-related gene sets to identify BCAT2 as a candidate target for verbascoside in NSCLC. Through a multi-level experimental approach—including molecular dynamics simulations, cell heat displacement assays, gain-of-function and loss-of-function experiments, and xenograft tumor models— we systematically validated the direct binding, biological function, and downstream mechanisms of BCAT2 as a core functional target.

The selection of BCAT2 as the primary study object was based on three main reasons. First, molecular docking showed that the binding energy of verbascoside to BCAT2 (−7.8 kcal/mol) was significantly lower than that of PARP1 (−4.42 kcal/mol), indicating a more stable and higher affinity binding to BCAT2. Second, PARP1 is a mature target whose mechanisms have been extensively studied [14], for example, Pan et al. found that high PARP1 activity in NSCLC is associated with poorer patient prognosis. PARP1-knockout NSCLC cells exhibit lower cisplatin resistance [15]. In contrast, the role of BCAT2 in regulating cell death in NSCLC, particularly ferroptosis, remains unclear. Only a study published in 2016 demonstrated that the loss of BCAT2 impairs tumor formation in NSCLC [16]. Therefore, exploring its potential as a novel verbascoside target is highly innovative. Third, BCAT2, as a core metabolic enzyme, directly affects mitochondrial function and redox homeostasis, which aligns more closely in pathophysiological logic with the observed mitochondrial damage, ROS accumulation, and ferroptosis phenotypes [17].

As a key rate-limiting enzyme in the first step of branched-chain amino acid (BCAA) catabolism, a growing body of evidence suggests that BCAA metabolism mediated by BCAT2 supports macromolecular biosynthesis and redox homeostasis in cancer cells and promotes tumor progression in colorectal, pancreatic, and prostate cancers [5,18,19]. However, little is known about the expression patterns and biological functions of BCAT2 in NSCLC. We first confirmed that BCAT2 is significantly upregulated in NSCLC tissues and various NSCLC cell lines, both at the mRNA and protein levels. Functional experiments demonstrated that BCAT2 overexpression enhances cell survival and inhibits apoptosis and ferrocytosis, whereas BCAT2 knockdown produces the opposite effects, supporting a pro-tumor role for BCAT2 in NSCLC at the cellular level. Furthermore, through molecular docking, molecular dynamics simulations, and CETSA assays, we provided direct evidence for the physical interaction between verbascoside and BCAT2, revealing that verbascoside reduces the thermal stability of the BCAT2 protein and inhibits its enzymatic activity.

Similarly, we found that inhibition of BCAT2 via verbascoside or shRNA-mediated knockdown leads to the accumulation of BCAAs and BCKA within cells. BCATs catalyze the breakdown of BCAAs into BCKAs, which can subsequently be reaminated by BCATs or oxidized into products of the tricarboxylic acid (TCA) cycle [12,20]. Cancer cells, however, rely on the metabolic breakdown of BCAAs to sustain their growth. BCAT2 deficiency leads to disruption of the tricarboxylic acid (TCA) cycle, which may reduce NADPH production, resulting in GSH depletion and the accumulation of lipid peroxides [13], thereby causing severe mitochondrial damage, including elevated ROS levels, reduced ATP synthesis, collapse of the mitochondrial membrane potential, and disruption of cristae ultrastructure, ultimately triggering ferrocytosis. This mitochondrial dysfunction subsequently activates endogenous apoptotic pathways, as evidenced by upregulation of the pro-apoptotic protein Bax, downregulation of the anti-apoptotic protein Bcl-2, and increased cleavage of caspase-3 [21]. The mitochondrial apoptotic pathway involves the regulation of mitochondrial outer membrane permeability by the Bcl-2 family of proteins. The dynamic balance between the pro-apoptotic protein Bax and the anti-apoptotic protein Bcl-2 acts as the “switch” for cell fate [22]. When Bax predominates, it forms pores in the mitochondrial membrane, thereby releasing apoptotic factors that activate initiator caspases and ultimately cleave caspase-3 to generate its active form, cleaved-caspase-3. As the final executor of apoptosis, the latter extensively cleaves cellular proteins, leading to cell death [23]. Therefore, the protein expression levels of Bax and Bcl-2 determine whether apoptosis is initiated, and the appearance of cleaved-caspase-3 marks the irreversible execution phase of apoptosis.

The anticancer activity of verbascoside has been supported by a large body of literature, and it has demonstrated some activity against prostate cancer, colorectal cancer, and ovarian cancer [24–26]. In NSCLC, we found that after verbascoside treatment or BCAT2 knockdown, typical biochemical changes of ferroptosis occurred within cells: depletion of GSH, accumulation of the lipid peroxidation end product MDA, Fe² ⁺ ion accumulation, accompanied by decreased expression of ferroptosis negative regulators, GPX4 and SLC7A11, and increased expression of the positive regulator, ACSL4. These changes closely match the known characteristics of ferroptosis [27]. Notably, this study found that mitochondrial dysfunction caused by BCAT2 inhibition, particularly the explosive accumulation of ROS and collapse of mitochondrial membrane potential, may be a key link between apoptosis and ferroptosis [28]. Excessive ROS can not only directly damage mitochondrial membranes and cristae structures (confirmed by TEM results), exacerbating the energy crisis (reduced ATP synthesis), but also directly oxidize polyunsaturated fatty acids in cell membranes, thereby initiating and amplifying ferroptosis [29]. Therefore, by inhibiting BCAT2, verbascoside may first disrupt cellular redox homeostasis and mitochondrial function, simultaneously initiating both apoptosis and ferroptosis—two death pathways that are independent yet intersecting—producing a synergistic antitumor effect. The ability of BCAT2 to regulate mitochondrial function has been confirmed in heart failure [30], and bioinformatic analysis to predict prognosis and construct risk models related to prostate cancer and mitophagy also includes the BCAT2 gene [31]. These study findings suggest that BCAT2 directly regulates mitochondrial function. Of course, this is only our speculation based on the existing results and requires further experimental validation. Furthermore, the phenomenon of verbascoside-inducing dual cell death is consistent with other reports on natural products, such as agrimonolide [32], alkannin [33], and agitinin C [34], which also induce apoptosis and ferroptosis, consistent with the trend observed in our study.

Although this study has achieved a series of meaningful findings, there are some limitations that should be addressed in future studies. First, this study focused on apoptosis and ferroptosis; however, the cell death modalities were diverse (e.g., autophagy and necroptosis). Whether verbascosides act via these pathways warrant further investigation. Second, the conclusions of this study are primarily based on cell and animal models. The safety and efficacy of the verbascoside-targeting-BCAT2 therapeutic strategy in patients with NSCLC still requires validation through rigorous pre-clinical pharmacokinetic studies and clinical trials.

## Conclusion

This study systematically demonstrated an innovative mechanism by which verbascoside, a novel BCAT2 inhibitor, induces dual apoptosis and ferroptosis in NSCLC cells by disrupting mitochondrial homeostasis, thereby providing important experimental evidence and a promising candidate molecule for the development of new natural product-based combination therapies for NSCLC.

## Supporting information

S1 Table Primer sequences for lentivirus – mediated over – expression and knockdown of BCAT2. (DOCX)

S2 Table Antibody information for western blot experiment. (DOCX)

S1 Fig Validation of BCAT2 overexpression and knockdown efficiency in NSCLC cell lines. (TIF)

S2 Fig Rescue assays validate the regulatory role of BCAT2 in ferroptosis of NSCLC cells. (TIF)

S3 Fig Effects of verbascoside on PARP1 cleavage and the DNA damage marker γ-H2AX in NCI-H1299 cells. (TIF)

## Abbreviations

NSCLC: non-small cell lung cancer
BCAT2: branched-chain amino acid transaminase 2
PARP1: poly(ADP-ribose) polymerase 1
RT-qPCR: reverse transcriptase quantitative polymerase chain reaction
CCK-8: cell counting kit-8
PI: propidium iodide
FITC: fluorescein isothiocyanate
GSH: reduced glutathione
GSSG: oxidized glutathione disulfide
MDA: malondialdehyde
ELISA: enzyme-linked immunosorbent assay
ROS: reactive oxygen species
DEGs: differentially expressed genes
FDR: false discovery rate
PBS: phosphate-buffered saline
TEM: transmission electron microscopy
GAPDH: glyceraldehyde-3-phosphate dehydrogenase
