# Supplementary material for: Verbascoside triggers apoptosis and ferroptosis in NSCLC by targeting BCAT2
Source: PLoS One. 2026 Jul 30;21(7):e0354955. doi: 10.1371/journal.pone.0354955 (PMC13422841; doi:10.1371/journal.pone.0354955)
Supplement: S1 Table — (DOCX) [file pone.0354955.s001.docx]

| Primer name | Primer sequences (5’ - 3’) |
| --- | --- |
| BCAT2 -target1-F | CCGGGGCTCATCGAAGTGGACAAGGCTCGAGCCTTGTCCACTTCGATGAGCCTTTTT |
| BCAT2 -target1-R | AATTAAAAAGGCTCATCGAAGTGGACAAGGCTCGAGCCTTGTCCACTTCGATGAGCC |
| BCAT2 -target2-F | CCGGCGATCACCATGAAGCAGTTGCCTCGAGGCAACTGCTTCATGGTGATCGTTTTT |
| BCAT2 -target2-R | AATTAAAAACGATCACCATGAAGCAGTTGCCTCGAGGCAACTGCTTCATGGTGATCG |
| BCAT2 -target3-F | CCGGCCGTGTTAGTGCAACAGGAGGCTCGAGCCTCCTGTTGCACTAACACGGTTTTT |
| BCAT2 -target3-R | AATTAAAAACCGTGTTAGTGCAACAGGAGGCTCGAGCCTCCTGTTGCACTAACACGG |
| ShNC-F | CCGGGTTCTCCGAACGTGTCACGTCTCGAGACGTGACACGTTCGGAGAACTTTTT |
| ShNC-R | AATTAAAAATTCTCCGAACGTGTCACGTCTCGAGACGTGACACGTTCGGAGAAC |
| pLV-CMV-BCAT2-F | AATGTACAAGGAATTCGCCACCATGGCCGCAGCCGCTCTGG |
| pLV-CMV-BCAT2-R | AATTATCTAGGGATCCTCACACCGGGAACATCCAC |

**S1 Table. Primer sequences for lentivirus - mediated over - expression and knockdown of BCAT2**
