# Supplementary material for: Verbascoside triggers apoptosis and ferroptosis in NSCLC by targeting BCAT2
Source: PLoS One. 2026 Jul 30;21(7):e0354955. doi: 10.1371/journal.pone.0354955 (PMC13422841; doi:10.1371/journal.pone.0354955)
Supplement: S2 Table — (DOCX) [file pone.0354955.s002.docx]

**S2 Table. Antibody Information for Western Blot Experiment**

| Gene Name | Manufacturer | Catalog Number | Suggested Western blot Dilution |
| --- | --- | --- | --- |
| ACSL4 | Affinity Biosciences | DF12141 | 1:1000 |
| BCAT2 | Affinity Biosciences | DF12851 | 1:1000 |
| GPX4 | Affinity Biosciences | DF6701 | 1:1000 |
| SLC7A11 (xCT) | Affinity Biosciences | DF12509 | 1:1000 |
| Bax | Affinity Biosciences | AF1020 | 1:1000 |
| Bcl-2 | Affinity Biosciences | AF6139 | 1:1000 |
| Caspase-3 | Affinity Biosciences | AF6311 | 1:1000 |
| Cleaved-Caspase3 | Affinity Biosciences | AF7022 | 1:1000 |
| Goat Anti-Rabbit IgG (H+L) HRP | Affinity Biosciences | S0001 | 1:5000 |
| GAPDH | Affinity Biosciences | AF7021 | 1:1000 |
