# Supplementary figures and images for: Verbascoside triggers apoptosis and ferroptosis in NSCLC by targeting BCAT2
Source: PLoS One. 2026 Jul 30;21(7):e0354955. doi: 10.1371/journal.pone.0354955 (PMC13422841; doi:10.1371/journal.pone.0354955)

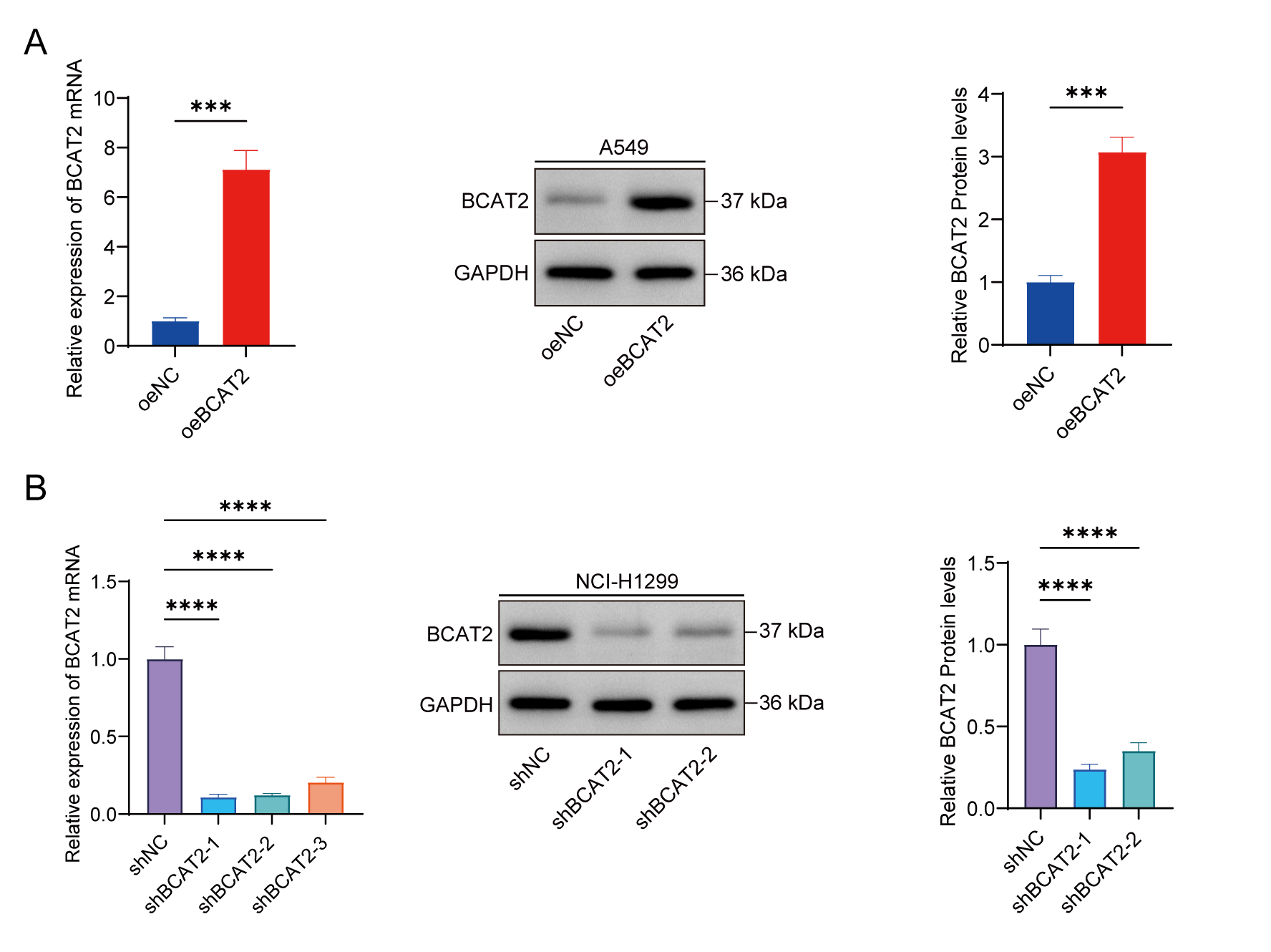

Supplement: S1 Fig — (TIF) [file pone.0354955.s003.tif]

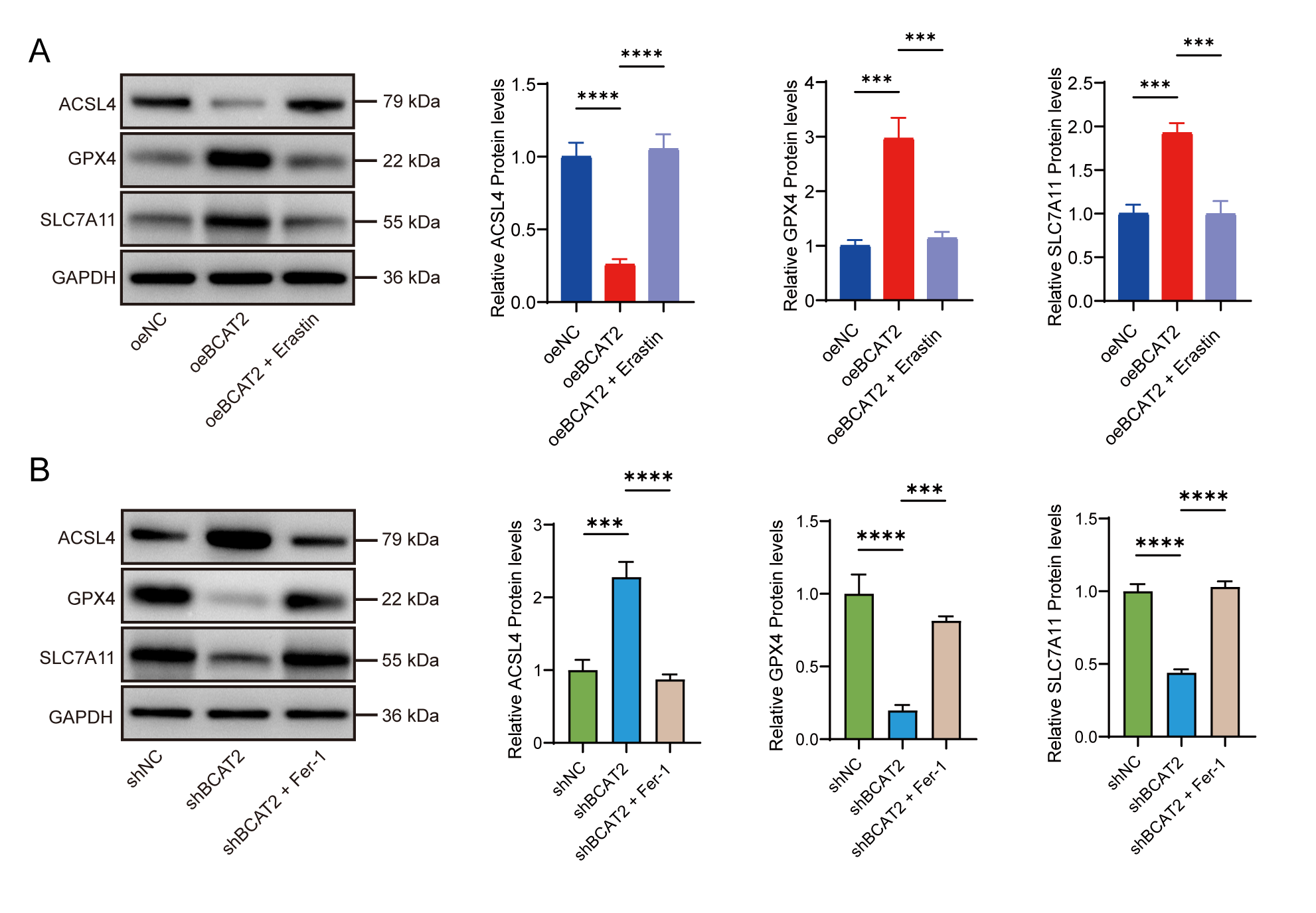

Supplement: S2 Fig — (TIF) [file pone.0354955.s004.tif]

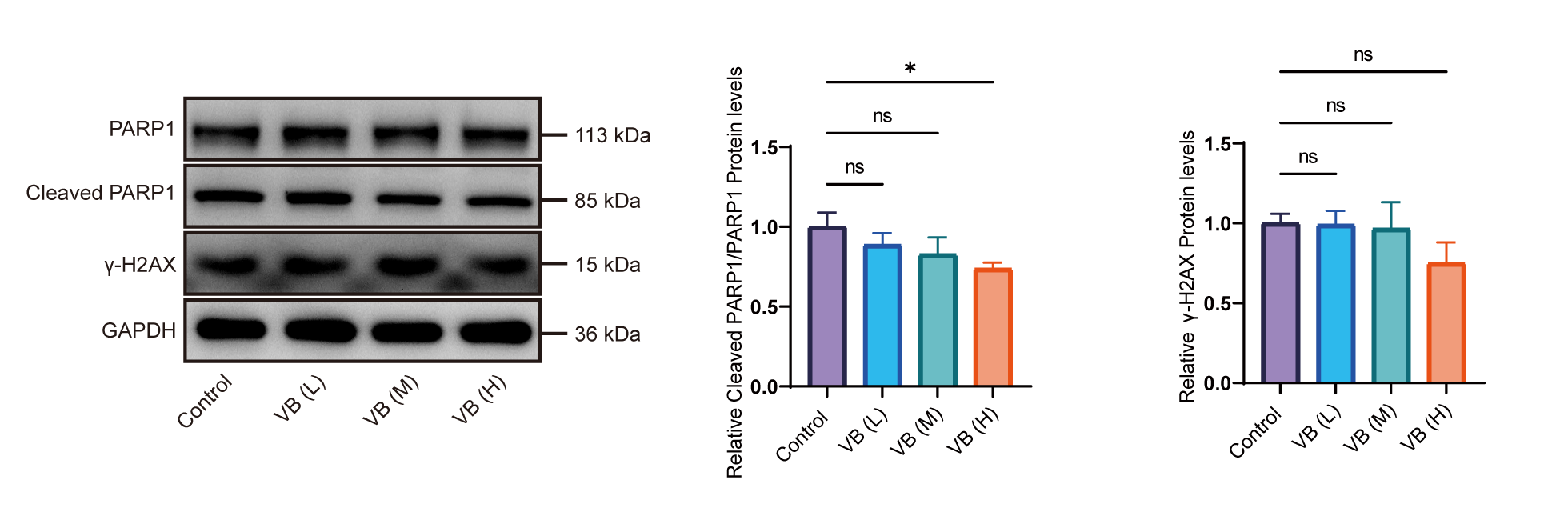

Supplement: S3 Fig — (TIF) [file pone.0354955.s005.tif]
